# Production, Validation, and Exposure Dose Measurement of [^13^N]Ammonia Under Academic Good Manufacturing Practice Environments

**DOI:** 10.3390/pharmaceutics17050667

**Published:** 2025-05-19

**Authors:** Katsumi Tomiyoshi, Yuta Namiki, David J. Yang, Tomio Inoue

**Affiliations:** 1 Shonan Research Institute of Innovative Medicine, Shonan Kamakura General Hospital, 1370-1, Okamoto, Kamakura 247-8533, Japan; y_namiki@shonankamakura.or.jp; 2 Advanced Medical Center, Shonan Kamakura General Hospital, Kamakura 247-8533, Japan; dj-yang@msn.com (D.J.Y.); t_inoue@shonankamakura.or.jp (T.I.)

**Keywords:** [^13^N]NH_3_, production, validation, exposure rate, academic GMP

## Abstract

**Objective:** Current good manufacturing practice (cGMP) guidance for positron emission tomography (PET) drugs has been established in Europe and the United States. In Japan, the Pharmaceuticals and Medical Devices Agency (PMDA) approved the use of radiosynthesizers as medical devices for the in-house manufacturing of PET drugs in hospitals and clinics, regardless of the cGMP environment. Without adequate facilities, equipment, and personnel required by cGMP regulations, the quality assurance (QA) and clinical effectiveness of PET drugs largely depend on the radiosynthesizers themselves. To bridge the gap between radiochemistry standardization and site qualification, the Japanese Society of Nuclear Medicine (JSNM) has issued guidance for the in-house manufacturing of small-scale PET drugs under academic GMP (a-GMP) environments. The goals of cGMP and a-GMP are different: cGMP focuses on process optimization, certification, and commercialization, while a-GMP facilitates the small-scale, in-house production of PET drugs for clinical trials and patient-specific standard of care. Among PET isotopes, N-13 has a short half-life (10 min) and must be synthesized on site. [^13^N]Ammonia ([^13^N]NH_3_) is used for myocardial perfusion imaging under the Japan Health Insurance System (JHIS) and was thus selected as a working example for the manufacturing of PET drugs in an a-GMP environment. **Methods:** A [^13^N]NH_3_-radiosynthesizer was installed in a hot cell within an a-GMP-compliant radiopharmacy unit. To comply with a-GMP regulations, the air flow was adjusted through HEPA filters. All cabinets and cells were disinfected to ensure sterility once a month. Standard operating procedures (SOPs) were applied, including analytical methods. Batch records, QA data, and radiation exposure to staff in the synthesis of [^13^N]NH_3_ were measured and documented. **Results:** 2.52 GBq of [^13^N]NH_3_ end-of-synthesis (EOS) was obtained in an average of 13.5 min in 15 production runs. The radiochemical purity was more than 99%. Exposure doses were 11 µSv for one production run and 22 µSv for two production runs. The pre-irradiation background dose rate was 0.12 µSv/h. After irradiation, the exposed dosage in the front of the hot cell was 0.15 µSv/h. The leakage dosage measured at the bench was 0.16 µSv/h. The exposure and leakage dosages in the manufacturing of [^13^N]NH_3_ were similar to the background level as measured by radiation monitoring systems in an a-GMP environments. All QAs, environmental data, bacteria assays, and particulates met a-GMP compliance standards. **Conclusions**: In-house a-GMP environments require dedicated radiosynthesizers, documentation for batch records, validation schedules, radiation protection monitoring, air and particulate systems, and accountable personnel. In this study, the in-house manufacturing of [^13^N]NH_3_ under a-GMP conditions was successfully demonstrated. These findings support the international harmonization of small-scale PET drug manufacturing in hospitals and clinics for future multi-center clinical trials and the development of a standard of care.

## 1. Introduction

Current good manufacturing practice (cGMP) guidance for PET drugs has been adopted by the European Medicines Agency (EMA) and the U.S. Food and Drug Administration (FDA) as standard manufacturing regulations. To support the development of innovative and effective PET drugs, researchers rely on the engagement with regulatory agencies to comply with chemistry, manufacturing, and controls (CMC) requirements. The CMC section of cGMP regulations includes batch records of the testing schedules (ingredients, strength, specific activity, purity, osmolality, pH, sterility, and pyrogenicity), new devices, facility qualification, certification, personnel, and audits by regulatory authorities.

In Japan, the Ministry of Health, Labor and Welfare (MHLW) is responsible for the approval of pharmaceutical and medical devices and provides regulatory guidance. The Pharmaceutical and Medical Devices Agency (PMDA), in collaboration with the MHLW, conducts scientific reviews to ensure the safety, efficacy, and quality of medical products to be marketed in Japan. At present, cGMP compliance is only required for radiopharmaceuticals intended for commercial distribution. For the in-house manufacturing of PET drugs within hospitals and clinics, there is no regulatory approval for cGMP compliance. Instead, radiosynthesizers are subject to regulatory approval as medical devices for in-house manufacturing of PET drugs in clinical services and clinical trials. These devices must ensure appropriate quality, sufficient safety, and efficacy in clinical practice [1]. Radiosynthesizers not approved by PMDA may only be used for clinical research or clinical trials. The manufacturing processes of PET drugs are neither under the regulatory authorities nor supervised by the vendors of radiosynthesizers. Therefore, hospitals and clinics are responsible for the in-house production of PET drugs.

To bridge the gap between radiochemistry standardization and site qualification, the JSNM launched an academic GMP (a-GMP) guidance for in-house PET drug manufacturing, imaging standardization, and site qualification [https://jsnm.org/english/, accessed on 19 March 2017]. Several institutes have implemented a-GMP setups in their radiopharmacy units [2,3,4,5,6,7]. While a-GMP shares documentation standards with cGMP to ensure quality for clinical research and care protocols, their goals differ: cGMP aims to optimize processes for commercialization, whereas a-GMP supports small-scale production for clinical use or innovative research. In Japan, a-GMP facilities are audited by designated auditing organizations to ensure compliance with JSNM standards.

PET radiopharmaceuticals enable molecular and metabolic imaging by targeting specific biological pathways. PET scanners combined with computed tomography (CT) allow for better delineation of tumor volumes and the determination of pharmacokinetics, pharmacodynamics, and radiation dosimetry through real-time and multi-slice imaging [8,9]. Reconstructed 3-dimensional whole-body images and lesion activity detected by PET/CT can assess the safety and efficacy of PET drugs [10,11]. PET is particularly valuable for cancer detection, neurological and cardiovascular imaging applications due to its superior resolution, sensitivity, and quantitative capabilities [12].

The most common organic radioisotopes used to label tracers by PET are carbon-11 (C-11, 20 min half-life), nitrogen-13 (N-13, 10 min half-life), oxygen-15 (O-15, 2 min half-life), fluorine-18 (F-18, 109 min half-life), and iodine-124 (I-124, 4.2-day half-life). These isotopes are used to label compounds for imaging tumors, neuronal disorders, and cardiovascular systems [13,14,15,16]. PET radiosynthesis must be rapid due to the short half-lives of the radioisotopes used. These isotopes decay and emit positrons, which annihilate with electrons, producing two gamma rays with a specific energy of 511 keV over time. With robust purification processes during synthesis, it is challenging to maximize the amount of a PET drug available for imaging. Thus, it is necessary to use radiosynthesizers manufacture PET drugs to avoid the higher risk of gamma radiation exposures during radiosynthesis. Among these radioisotopes, F-18, C-11, and I-124 have been the subject of commercial interest due to their longer half-lives. They can be manufactured in an industrial cGMP facility and delivered to hospitals. N-13, and O-15 have great values to assess metabolic pathways with minimal structural alterations; however, they lack commercial interest due to their too-short half-lives. Thus, PET drugs labeled with N-13 and O-15 require in-house manufacturing using radiosynthesizers. In this study, we focus on the in-house synthesis of the PET tracer [^13^N]ammonia ([^13^N]NH_3_) using a radiosynthesizer in an a-GMP environment.

Ammonia (NH_3_), nonionic form, is involved in the pathways of glutamic acid [17], glutamine [18], carbamyl phosphate [19], purines [20], and urea [21], and is rapidly permeable to all cell membranes through blood flow systems. Given its high myocardial extraction and retention, [^13^N]NH_3_ is a useful PET drug to evaluate myocardial perfusion imaging (MPI) under rest or pharmacologic stress conditions in patients with known or suspected coronary artery disease (CAD) [22]. PET-MPI with [^13^N]NH_3_ offers high sensitivity and overall accuracy for detecting CAD [22]. [^13^N]NH_3_ was also shown to be useful in imaging brain and liver tumors [15,23,24].

In Japan, [^13^N]NH_3_ has been practiced for MPI and covered under the Japan Health Insurance System (JHIS) since 2012. PMDA authorized radiosynthesizers (N-100 by Sumitomo Heavy Industries, Ltd.; ammonia synthesizer by JFE Engineering Co., Japan) dedicated to [^13^N]NH_3_. Thus, [^13^N]NH_3_ was selected as a working example for the in-house production of PET drugs in our hospital under a-GMP regulations.

In this study, we report on the quality assurance (QA), product validation, equipment for monitoring the personnel exposures, and air quality for the in-house production of [^13^N]NH_3_ in an a-GMP compliant facility. The in-house production of PET drugs in a-GMP environments offers standardized processes for producing drugs in these settings, which should improve the safety and efficacy for patients and provide efficiency in clinical practice.

## 2. Method and Materials

### 2.1. [13N]NH_3_ Production Using an Automated Module N100 System

Radiochemical preparation was performed in Hot Lab 2, an a-GMP-compliant laboratory equipped for small-scale automated radiopharmaceutical production at our institution. [^13^N]NH_3_ was produced on-site with the HM-12S Cyclotron System Sumitomo Heavy Industries, Ltd.,(Tokyo, Japan). The in-target production of [^13^N]NH_3_ was achieved by adding free radical scavengers (ethanol) into the target water to prevent the formation of the OXO anions of nitrogen (^13^NO_3_^−^ and ^13^NO_2_^−^). This method, reported by Wieland et al. [25] and Berridge et al. [26], demonstrated that ethanol effectively enhances the [^16^O(p,α)^13^N] nuclear reaction using cyclotrons of energies >10 MeV for the production of [^13^N]NH_3_. The O-16 enriched (99.99%) target water was bought from Otsuka Pharmaceutical Co. Ltd. (Tokyo, Japan). Ethanol in water (10 mmol/L) was obtained from FUJIFILM Wako Pure Corp (Osaka, Japan). The clumn (Accel CM) used to trap ammonia was purchased from Waters (Milford, MA, USA).

**Figure 1:**
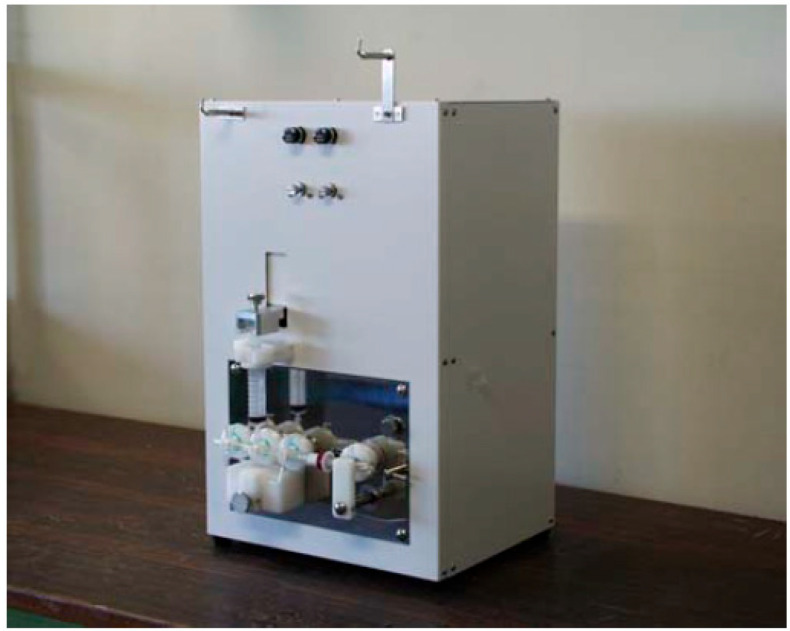
N100 [^13^N]NH_3_ synthesizer.

**Figure 2:**
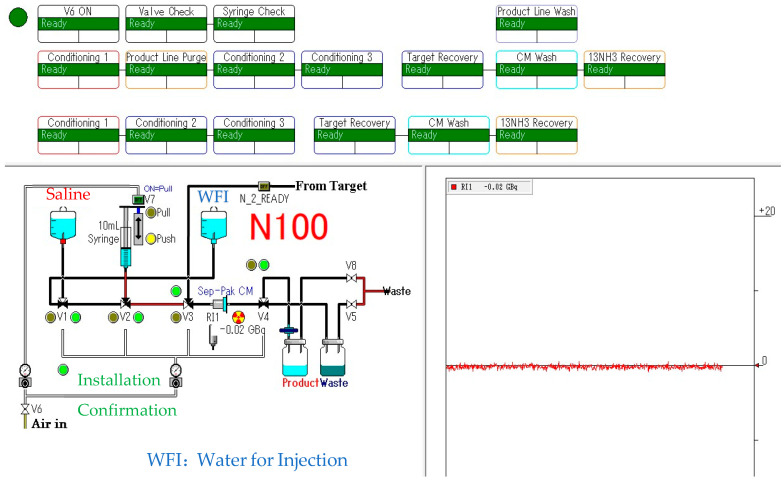
[^13^N]NH_3_ synthesis block diagram.

[^13^N]NH_3_ was synthesized using an automated module N100 Sumitomo Heavy Industries, Ltd. (Tokyo, Japan), which was approved by PMDA as a medical device (Figure 1). Its software block diagram is shown in Figure 2. Irradiation time for synthesis was fixed at 5 min with a beam current of 50 μA. The target was loaded with 10 mmol/L of ethanol in water for production via the nuclear reaction ^16^O(p,α)^13^N. The target water was introduced into an Accel CM column that trapped [^13^N]NH_3_. The column was eluted with injectable water and then drained with physiological saline to obtain a drug product as an injectable drug [27,28,29,30,31].

### 2.2. Quality Assurance (QA) Measurements

a-GMP regulations require [^13^N]NH_3_ to be subjected to a single QA test at the first run. QA is waived for subsequent syntheses. The criteria of QA for the first run and for three consecutive lots of [^13^N]NH_3_ with their radionuclide purity and identity (half-life), radiochemical purity, pH, endotoxin(EU/mL), and sterility levels are summarized in Table 1.

Radiochemical purity was determined using a high-performance liquid chromatography (HPLC) system LC-20A Shimadzu (Kyoto, Japan). The analytical column used was a Nova-Pak C18 (4 µm 3.9 mm × 150 mm) with the mobile phase consisting of PIC B8 0.005M/acetonitrile (4:6) at a flow rate of 1 mL/min. The UV (280 nm) and radioisotope (RI) detectors were obtained from Shimadzu and Universal Giken (US-3300), respectively. Radionuclide identity testing was carried out using a Multi-Channel Analyzer (MCA) DS-P1000 Spectrum Station SEIKO EG and G (Tokyo, Japan). Other tests, such as the formaldehyde test, sterility test, and endotoxin test, were conducted as follows. Formaldehyde was tested using a formaldehyde color reagent, PACK TEST Kyoritsu Chemical Lab., Corp.( Yokohama, Japan). The sterility test was performed by incubating [^13^N]NH_3_ in both a thioglycol liquid medium and an SCD liquid medium (Darmstadt, German) in an A3001 incubator Ikuta Sangyo, (Aichi, Japan). The endotoxin test was performed using a Toxinometer ET-7000 with Limulus ES-II plus CS single Test Wako299-77201 Fujifilm Wako Pure Chemical Corporation (Osaka, Japan).

### 2.3. Exposure Dose and Leakage Dose Measurements in [^13^N]NH_3_ Synthesis

[^13^N]NH_3_ synthesis was carried out in Hot Lab 2 by two personnel: a cyclotron operator and a staff member responsible for the automated synthesis module. Five consecutive syntheses were sufficient to provide doses for two patients. The patients received [^13^N]NH_3_ for both rest and stress imaging. Exposure dose and leakage dose were routinely measured in Hot Lab 2. The exposure dose was measured using a certified Aloka pocket dosimeter (Musashino, Tokyo). The leakage doses of [^13^N]NH_3_ were measured using an area monitor ((Musashino, Tokyo) of 11 syntheses runs before and synthesis time in Hot Lab 2. The leakage doses were measured in front of the cell during synthesis and at a computer desk, which was 1 m away from the cell.

### 2.4. a-GMP Environment Measurements

The conditions in a-GMP that meet standard values for a PET drug manufacturing environment are as follows.

(1) Work areas (hot labs, dispensing rooms, and quality testing rooms) must maintain Class C cleanliness for environmental particles (≧0.5 µm) and microorganisms.
(2) The hot cell of the closed-system synthesis equipment must meet Class A cleanliness standards.

**Figure 3:**
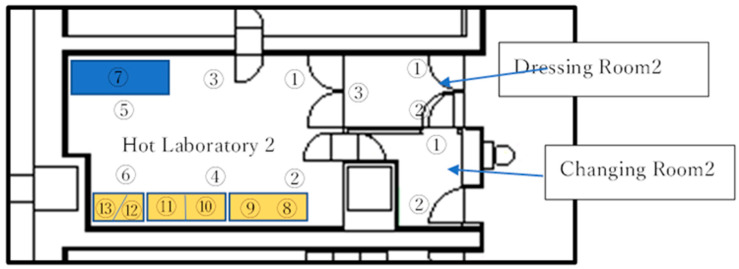
Each number designated the position for measurements on the Hot Lab 2 Floor. Numbers 8–13 were the site for hot cells.

These examinations and all tests were carried out for 3 batches. The cleanliness of Class A and Class C of airborne and adherent bacteria is illustrated in Table 2. An example of cleanliness for Class A and Class C of airborne particulate levels (particles/m^3^) in Hot Lab 2 is shown in Table 3 and Figure 3.

## 3. Results and Discussion

### 3.1. [13N]NH_3_ Production Using an Automated Module N100 System

Five [^13^N]NH_3_ syntheses were conducted. Each synthesis was divided into three batches, for a total of fifteen synthetic lots. The reason for selecting five syntheses conducted in one day was that the dosages were sufficient for two patients in addition to satisfying the criteria for QA of [^13^N]NH_3_. After irradiating the target for 5 min, an average of 2.52 GBq of [^13^N]NH_3_ was obtained in 13.5 min for the end of synthesis (EOS). Table 4a,b displays the time results for cyclotron irradiation, product EOS, and yield across five syntheses and fifteen tests.

The vial containing the drug product for clinical use was finally lifted into the inspection room using a baggage lift. The total time was within 14 min. [^13^N]NH_3_ was then shipped quickly to the patients for their testing at rest and under stress during diagnosis of CAD. Two syntheses were required for CAD studies. The first run studied the imaging for the rest test. The second run studied imaging for a stress test. Each patient’s rest or stress process took about 13 min from synthesis to shipment. The rest of the stress imaging took about 20 min.

### 3.2. QA of [^13^N]NH_3_

**Figure 4:**
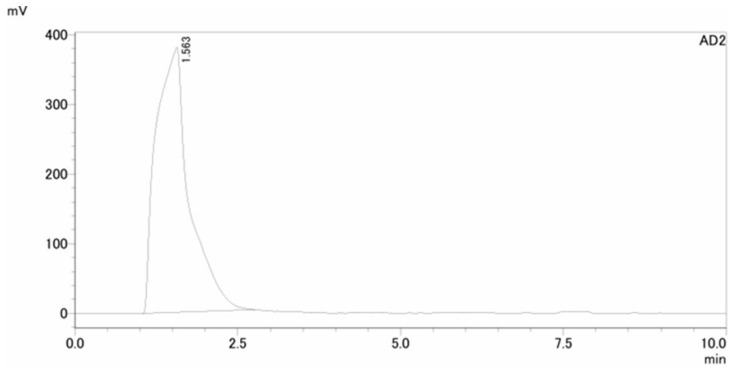
HPLC spectrum of [^13^N]NH_3_ for radiochemical purity (>99%).

**Figure 5:**
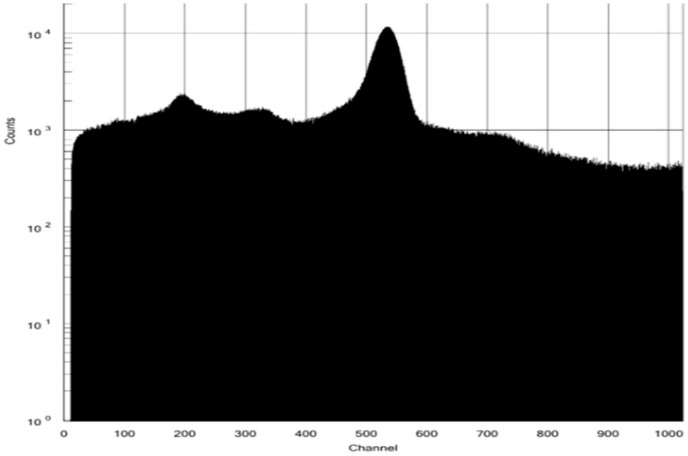
MCA spectrum for N-13 radionuclide identity and purity.

The QA of [^13^N]NH_3_ includes radionuclide identity, radiochemical purity, and half-life testing with sufficient radioactivity in the timeframe assessment. The retention time of [^13^N]NH_3_ in the HPLC spectrum was about 1.5 min. No additional peaks were observed, indicating a radiochemical purity greater than 99% (Figure 4). A peak at 0.511 MeV in the MCA spectrum confirmed the radionuclide identity of N-13, with a radionuclide purity exceeding 99% (Figure 5). A decay correction method would be required if the activity is too low because it ensures accurate measurements by accounting for the natural decay of radioactive materials over time. Other than sterility testing, all 11 items, including tests for endotoxin and formaldehyde that were completed within one hour, satisfied the quality testing conditions (shown in Table 1). Sterility testing requires 14 days for the cultivation of anaerobic and aerobic bacteria. There were no bacteria found in any tests to date.

### 3.3. Exposure Dose and Leakage Dose Measurements

#### 3.3.1. Exposure Dose Measurements

**Figure 6:**
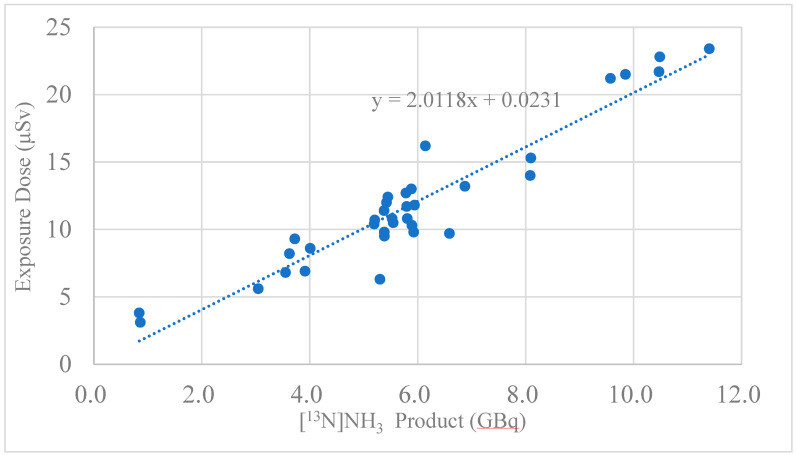
Correlation between [^13^N]NH_3_ product (GBq) and exposure dose (μSv).

The radiation exposure doses for the cyclotron operator and the synthesis staff using an automated module are presented in Table 5a,b. Table 5a shows the result of the exposure doses from two batches of production runs using an automated radiosynthesizer for one patient, and Table 5b shows the result of the dose exposures from four batches of production runs using an automated radiosynthesizer for two patients. The radiation exposure dose was almost proportional to the amount of synthesized radioactivity (Figure 6). The five-point radiation exposure doses of 20 μSv or more were collected from all five syntheses; the two points with radiation exposure doses of 5 μSv or less were synthesized twice, and the rest were synthesized three to four times. From these results, a slope k = 2 (μSv/GBq) was obtained through the origin, and a radiation exposure dose of 2 μSv per GBq was obtained.

The annual radiation dose limit for individual members of the public is generally 1 mSv per year, as set by the Nuclear Regulatory Commission (NRC), and the limit for exposure in unrestricted areas should not exceed 0.02 mSv in any one hour. As shown in Table 5a,b, the exposure dose was clearly different between the cases of one patient and two patients, with the dose being around 11 µSv for one patient and 22 µSv for two patients, which was about twice as much. This implies that exposure per staff member for two patient runs may approach regulatory limits. The cause of this exposure to staff was thought to be that the product was taken out of the cell, placed in front of the camera for the measurement of liquid volume, and the staff was then exposed when it was moved to the mobile shielded container. Radiation exposure of the radiosynthesis staff was re-examined for their health condition three months after the initiation of [^13^N]NH_3_ radiosynthesis. On the other hand, the average exposure dose to the cyclotron operator was 2.83 ± 2.45 μSv, which was below the limit of exposure dose. Although present in the same hot lab, the operator did not handle the product directly and mainly operated a computer.

#### 3.3.2. Leakage Dose Measurements

**Figure 7:**
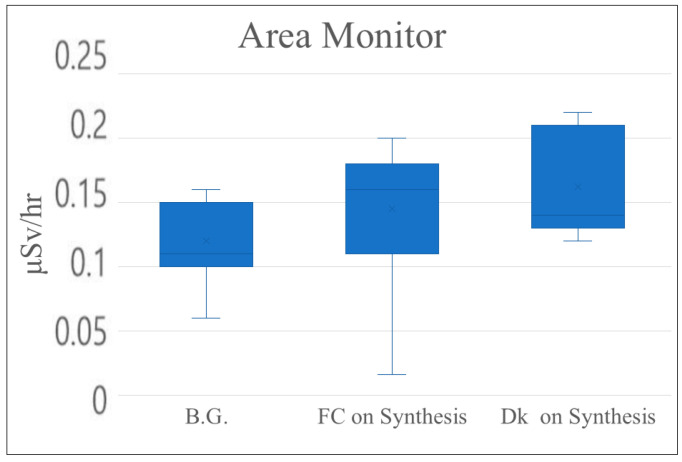
Leakage dose (mSv/h) with area monitor (B.G.: background; FC: front hot cell; Dk: computer desk).

Exposure and leakage doses were measured using an area monitor. Background measurements prior to irradiation were 0.12 μSv/h (background). Post-irradiation values were 0.15 μSv/h in front of the hot cell and 0.16 μSv/h at the computer desk, as shown in Table 6 and Figure 7. The area monitor was installed on the wall in the center of the room and did not change depending on the location, so the leakage dose before and after irradiation was thought to be within the measurement error.

### 3.4. An a-GMP Environment Measurements

We performed environmental measurements of airborne bacteria, adherent bacteria, and airborne particles in both the hot cell and the room of Hot Lab 2, as illustrated in Figure 3. The measurement results for the hot cell are shown in Table 7, while data for hot lab2 are shown in Table 8.

From August 2022 to May 2023, neither the hot cell nor Hot Lab 2 met the a-GMP environmental standards (Table 7 and Table 8). The issue was traced to insufficient airflow, with air moving in the wrong direction. The air direction flowed back from the corridor to Hot Lab 2. We then improved the air volume and quality by installing HEPA filters and prefilters. A routine sterilization protocol was implemented in Hot Lab 2 until the a-GMP environmental requirements were met. After these corrections, the a-GMP environmental conditions were met in November 2023. Radiosynthesis, QA testing, and site management have been carried out in the a-GMP environment since November 2023.

## 4. Conclusions

[^13^N]NH_3_ is a unique PET radiopharmaceutical used to assess metabolic pathways involved in the production of amino acids, purines, and urea [18,20,32]. Its clinical applications in rest and stress testing for the diagnosis of CAD are well documented [15,22,33]. However, the short half-life of N-13 (10 min) presents challenges for the synthesis and quality assurance of [^13^N]NH_3_ [27,29,31]. Thus, it is necessary to synthesize [^13^N]NH_3_ quickly using a radiosynthesizer in a clean environment, consistently conduct quality assurance, and manage radiation safety in an a-GMP environment.

In this study, we successfully established an a-GMP-compliant environment for the in-house manufacturing of PET drugs. The a-GMP facility requires documentation such as a flowchart of personnel assignments, systems to detect particulates in airflow, the use of radiosynthesizers within hot labs, adequate analytical equipment for the validation of radiopharmaceuticals, and a system to measure radiation exposure to the cyclotron operator and radiosynthesis staff. The JSNM provides the site audit. We found that the [^13^N]NH_3_ radiosynthesizer was able to offer batch-to-batch reproducibility. The CMC section for [^13^N]NH_3_ includes the identity and purity in N-13 determined by its half-life and photon peak in multiple channel analyzer; the appearance of the product in solution free from particulates; radiochemical purity, identity, and yield assessed by radio-TLC and HPLC equipped with radioactive detector; pH within the range 5.5–8.0; and specific activity (Ci/μmol) assessed using dose calibrator and radio-HPLC. In addition to chemical analysis, [^13^N]NH_3_ must meet safety criteria, including sterility and pyrogenicity assessed by the bacterial endotoxins test (BET) [34]. We were able to produce multiple doses for MPI, and the level of radiation exposure to personnel was below the limit in routine clinical practice. With the successful small-scale in-house production of [^13^N]NH_3_ under an a-GMP setting, our findings support international harmonization on clinical phase trials of PET drugs involving multiple centers.

## Figures and Tables

**Table 1:** Criteria of QA for [^13^N]NH_3_.

Test Items	Standard Value	Frequency
1 Item		
1-1 Volume per Bach (mL)	10 ± 1	After each synthesis
1-2 Half-Life (min)	9.5~10.5	After each synthesis
2 Properties		
2-1 Visual State	Colorless and Transparent	After each synthesis
2-2 Presence of Particles	Imperceptible	After each synthesis
3 Endotoxin Test	Less than 6.8 EU/mL	After each synthesis
4 Sterility Test	Imperceptible	After each synthesis
5 pH	5~8	After each synthesis
6 Nuclide test	Peak in 511 keV	More than once per year
7 Purity test		
7-1 Hetero Nuclide Test	511 keV and/or 1.02 MeV	More than once per year
7-2 Chemical Purity Test	More than 95%	After each synthesis
7-3 Formaldehyde	Less than 2 ppm	After each synthesis

**Table 2:** Cleanliness standard for Class A and Class C of airborne and adherent bacteria (CFU: Colony Forming Unit).

Grade	Airborne Bacteria (CFU)/Plate	Adherent Bacteria (CFU)/Glove
A	<1	<1
C	100	25

**Table 3:** Cleanliness standard for Class A and Class C of airborne particulates (particles/m^3^).

Grade	Airborne Particulates
A	Maximum 3520 particles ≧ 0.5 μm/m^3^
C	Maximum 352,000 particles ≧ 0.5 μm/m^3^

**Table 4:** (**a**) Cyclotron irradiation time and yield of the final product for a total of 15 runs in 3 batches. (**b**) Irradiation time, synthesis time, and total time. Color grey highlights the findings in each run.

(a)								
Iteration	1	2	3	4	5	6	7	8
Irradiation time (min)	5	5	5	5	5	5	5	5
CM Column (GBq)	2.78	2.71	2.73	2.67	2.64	2.53	2.73	2.82
Final product (GBq)	2.66	2.63	2.61	2.54	2.37	2.39	2.58	2.60
	**9**	**10**	**11**	**12**	**13**	**14**	**15**	**Avg.**
Irradiation time (min)	5	5	5	5	5	5	5	5
CM Column (GBq)	2.79	2.76	2.61	2.59	2.69	2.71	2.66	2.69
Final product (GBq)	2.54	2.53	2.49	2.42	2.48	2.52	2.46	2.52
(**b**)								
	**1**	**2**	**3**	**4**	**5**	**6**	**7**	**8**
Irradiation time (min)	5	5	5	5	5	5	5	5
Synthesis time (min)	10.72	8.50	8.03	9.05	9.27	8.00	8.35	8.40
Total time (min)	15.72	13.50	13.03	14.05	14.27	13.00	13.35	13.40
	**9**	**10**	**11**	**12**	**13**	**14**	**15**	**Avg.**
Irradiation time (min)	5	5	5	5	5	5	5	5
Synthesis time (min)	8.30	7.93	7.37	9.72	7.95	8.53	8.95	8.60
Total time (min)	13.30	12.93	12.37	14.72	12.95	13.53	13.95	13.60

**Table 5:** (**a**) The exposure dose (µSv) measurement for one patient for the working staff. (**b**) The exposure dose (µSv) measurement for two patients for the working staff.

(a)		
Date	Exposure Dose for Synthesis Staff	Exposure Dose for Machine Operator
20231205	11.8	--
20240313	6.3	--
20240410	10.4	--
20240424	9.8	0.9
20240522	9.5	1.0
20240529	11.4	1.1
20240710	10.7	0.8
20240723	11.7	1.6
20240807	13.0	0.5
20240820	12.7	1.5
20240828	10.8	1.0
Average	10.73 ± 1.83 (*n* = 11)	1.07 ± 0.39 (*n* = 8)
(**b**)		
**Date**	**Exposure Dose for Synthesis Staff**	**Exposure Dose for Machine Operator**
20240626	21.2	1.5
20240702	23.4	6.5
20240904	21.7	1.8
20240910	22.8	1.5
Average (*n* = 4)	22.27 ± 1.00	2.83 ± 2.45

**Table 6:** Leakage dose(μSv/h) with area monitor.

Area Monitor	Before Irradiation	Front Cell on Irradiation	On the Desk on Irradiation
1	0.11	0.15	0.15
2	0.06	0.02	0.13
3	0.1	0.12	0.12
4	0.15	0.16	0.21
5	0.12	0.18	0.19
6	0.11	0.11	0.14
7	0.1	0.18	0.14
8	0.11	0.11	0.14
9	0.15	0.20	0.22
10	0.15	0.20	0.22
11	0.16	0.17	0.12
Average (n = 11)	0.12 ± 0.03	0.15 ± 0.05	0.16 ± 0.04

**Table 7:** Class A test for Hot Cell at Hot Lab 2 (Exc.: except).

**Measurement Date**		**2022/8/~2023/5/**	**2023/11/**	**2024/** **9** **/**	**2025/1/**
Airborne bacteria		0~10	0	0	0
Adherent bacteria	Upper Cell	0~5	0	0	0
	Lower Cell	0~4	0	0	0 Exc.⑨ = 1 Exc.⑪ = 1
Airborne particle	≧0.5	0~5	0~46	3~306	0~203 Exc.⑧ = 9721

**Table 8:** Class C test for Room at Hot Lab 2 (Exc.: except).

Measurement Date		2022/8/~2023/5/	2023/11/	2024/9/	2025/1/
Airborne bacteria		3~70	1~4	0~11	0~4
Adherent bacteria		11~∞	0~8	1~2	2~17 Exc. ③ = ∞ Exc. ⑥ = ∞
Airborne particle	≧0.5	202~1386	512~2846	140~742	1790~2712

## Data Availability

The data from the publication can be obtained by requesting it from the leading author, K.T.
